# The Mediating Effects of Diabetes Self‐Management on the Relationship Between Diabetes Distress and Quality of Life Among School‐Age Children With Type 1 Diabetes Mellitus During the COVID‐19 Pandemic

**DOI:** 10.1155/pedi/2022750

**Published:** 2026-02-19

**Authors:** Jiaxin Luo, Qingting Li, Yuwen Gao, Fang Liu, Jie Zhong, Ka Yan Ho, Robin Whittemore, Jia Guo

**Affiliations:** ^1^ Xiangya School of Nursing, Central South University, Changsha, Hunan, China, csu.edu.cn; ^2^ Department of Metabolism and Endocrinology, The Second Xiangya Hospital of Central South University, Changsha, China, csu.edu.cn; ^3^ Clinical Nursing Teaching and Research Section, The Second Xiangya Hospital of Central South University, Changsha, China, csu.edu.cn; ^4^ School of Nursing, The Hong Kong Polytechnic University, Hong Kong, China, polyu.edu.hk; ^5^ Health Sciences School of Nursing, Northeastern University Bouvé College, Boston, Massachusetts, USA; ^6^ School of Nursing, Yale University, New Haven, Connecticut, USA, yale.edu

**Keywords:** diabetes distress, diabetes self-management, quality of life, school-age children, type 1 diabetes

## Abstract

**Background:**

Diabetes distress is prevalent among youth with type 1 diabetes mellitus (T1DM) and can negatively impact their quality of life and metabolic control. Identifying modifiable factors to reduce this distress is crucial. This study investigates the interplay between diabetes distress, self‐management, and quality of life in school‐aged children with T1DM amidst the COVID‐19 pandemic. Its primary objective is to identify modifiable factors that can assist these children as they navigate the challenges associated with transitioning into adolescence.

**Methods:**

A cross‐sectional study with data from 341 Chinese school‐age children aged 8–12 was conducted. Data were collected through an online self‐report survey during the COVID‐19 pandemic (June–December 2022). The data included sociodemographic and clinical characteristics, diabetes distress, diabetes care activities and diabetes problem solving of diabetes self‐management and quality of life. Structural equation modeling assessed relationships and mediation effects.

**Results:**

All four domains of diabetes distress exhibited negative associations with quality of life (*r* = −0.74 to −0.77, *p* < 0.01). Care activities and problem‐solving related to diabetes self‐management mediated the associations of emotional burden and regimen‐related distress with quality of life (both *p* < 0.05). Conversely, neither diabetes care activities nor diabetes problem‐solving mediated the relationship between physician‐related distress and quality of life.

**Conclusions:**

Our findings indicate that problem‐solving techniques related to diabetes self‐management might be more effective at alleviating various aspects of diabetes distress—such as emotional burden, regimen‐related distress, and interpersonal distress—compared to diabetes care activities. Interventions that teach structured problem‐solving strategies could be beneficial. Given the ongoing pandemic, these findings could also serve as useful guidance for developing support strategies for school‐age children with chronic conditions during future public health emergencies.

## 1. Introduction

Type 1 diabetes mellitus (T1DM) is one of the most common chronic conditions during childhood [1]. It is estimated that 600,900 children under the age of 15 years live with this condition worldwide [2]. School‐age children with T1DM are more sensitive to a lack of insulin than adults and are therefore at a higher risk of rapidly developing acute diabetes‐related complications [3]. Quality of life, though qualitatively different from metabolic control, is increasingly recognized as an important psychosocial outcome among school‐age children with T1DM [4]. In both the United States and China, poorer quality of life has been consistently observed among school‐age children with T1DM than among their healthy peers [5, 6].

Several factors have been found to be associated with quality of life among school‐age children with T1DM. Sociodemographic characteristics such as older age, being born to a single‐child family, dropping out of school, and poor family economic condition have been associated with poorer quality of life [7–11]. Psychological variables such as high stress, high anxiety, increased depressive symptoms, poor peer relationships, and deficient family intimacy have also been identified as associated factors [5, 10, 12].

Diabetes distress, a distinct psychological factor, refers to the emotional burden, frustration, anxiety, worries, and stressors that arise from living with or managing this serious and complex condition [13]. Four domains of diabetes distress can be identified based on its origins: emotional burden, physician‐related distress, regimen‐related distress, and diabetes‐related interpersonal distress [14]. Unlike common psychological symptoms, such as stress and depressive symptoms, diabetes distress is often not adequately recognized by healthcare providers. A study conducted in Canada found that a higher level of diabetes distress was associated with lower quality of life among individuals with T1DM aged 16–17 years [15]. Moreover, a study conducted in Australia showed that elevated diabetes distress was one of the strongest predictors of poor metabolic control among adolescents with T1DM [16]. However, studies on diabetes distress among school‐age children with T1DM are lacking. Therefore, it remains unclear whether this relationship exists in this population.

According to the conceptual model of childhood adaptation to T1DM [4], health behaviors (e.g., diabetes self‐management) may play an important mediating role between psychological status (e.g., diabetes distress) and health outcomes (e.g., quality of life). T1DM self‐management, a widely acknowledged diabetes‐related behavioral variable, is defined as an active, daily, and flexible process whereby children and their parents share responsibility and decision‐making for achieving positive health outcomes [4]. This process encompasses collaboration with parents, diabetes‐related care activities, problem‐solving, communication, and setting goals [17]. A study of school‐age children aged 8–12 years found that better diabetes self‐management was associated with improved quality of life [18]. In another study of youth with T1DM aged 11–14 years, enhanced diabetes self‐management could alleviate the adverse impact of increased depressive symptoms on quality of life [19]. However, the potential mediating role of diabetes self‐management in the association between diabetes distress and quality of life remains underexplored. Understanding this relationship is critical to supporting school‐age children with T1DM, as it can inform appropriate pediatric nursing interventions aimed at improving their quality of life. Moreover, it may help to improve the transition to adolescence, a particularly difficult time for teens with T1DM.

Although current evidence and our model [4] suggest diabetes self‐management mediates the effect of diabetes distress on quality of life, more research is needed to confirm this, particularly in the context of COVID‐19, which may have heightened distress due to added stressors and healthcare disruptions. Therefore, this study aimed to explore the associations between the four domains of diabetes distress and quality of life and to examine whether care activities and problem‐solving related to diabetes self‐management mediates these relationships among Chinese school‐age children with T1DM during this period.

## 2. Methods

### 2.1. Design

This study adopted a nonexperimental, multicenter cross‐sectional survey design. The Strengthening the Reporting of Observational Studies in Epidemiology (STROBE) checklist was used to report the findings [20]. See Supporting Information: Supporting File 1.

### 2.2. Setting and Sample

This study was conducted at the Second Xiangya Hospital of Central South University, the First Hospital of Xinjiang Medical University, and the Sinocare Diabetes Foundation. To ensure statistical power in the mediation model, the sample size should be at least 30 times the number of predictors [21]. Based on prior research and clinical experience, 10 factors likely to influence the dependent variable (quality of life) were used as control variables (such as age, sex, diabetes duration, and glycosylated hemoglobin [HbA1c]) and independent variables (such as emotional burden, physician‐related distress, regimen‐related distress, diabetes‐related interpersonal distress, and diabetes care activities and problem‐solving as aspects of diabetes self‐management). Thus, a sample size of 300 individuals was required. To account for a potential online survey nonresponse rate of up to 20%, it was determined that 375 participants should be recruited. Thus, 375 school‐age children completed the online questionnaires. However, 34 participants were excluded from the analysis due to incorrect responses (e.g., all possible answers to an item selected). Thus, 341 (90.9%) participants were included in this study.

### 2.3. Inclusion and Exclusion Criteria

Inclusion criteria were: (1) age 8–12 years (school‐age per Varni et al. [22]), (2) T1DM diagnosis with ≥6 months of insulin therapy [23], and (3) Mandarin proficiency. Exclusion criteria included severe comorbidities (e.g., cancer and asthma).

### 2.4. Ethical Considerations

The study protocol was approved by the Ethics Review Board of Xiangya School of Nursing, Central South University (Number E201945) and followed the Declaration of Helsinki. Anonymous online surveys ensured confidentiality. Informed consent was obtained electronically from the parents or legal guardians of all participants under the age of 16. Parents reported sociodemographic data, while children completed psychosocial measures.

### 2.5. Measures

The data collected included sociodemographic and clinical characteristics, diabetes distress (independent variables), care activities and problem‐solving related to diabetes self‐management (mediating variables), and quality of life (dependent variable). Sociodemographic and clinical data were collected using a questionnaire developed by the research team. The sociodemographic data included age, sex, parental education levels, and annual family income. The clinical data included diabetes duration, HbA1c, and insulin treatment regimen. HbA1c levels were extracted from medical records to ensure accuracy.

Diabetes distress was assessed using the Chinese version of the Diabetes Distress Scale (DDS) [24], administered to children with standardized parental guidance to support comprehension of the items. Prior to the main study, we conducted a pilot test with 20 school‐aged children (ages 8–12) who met the inclusion criteria for T1DM diagnosis and regular attendance at diabetes clinic visits. The pilot was carried out in a clinical setting, where both children and their parents participated. Parents received a brief orientation session outlining the purpose of the DDS, instructions for providing neutral assistance, and strategies for clarifying item meanings without influencing responses. During administration, children completed the scale with their parents present to explain unfamiliar terms or concepts, following the standardized guidance protocol. Feedback was collected through structured interviews with both children and parents, focusing on item clarity, perceived relevance, and the degree of parental involvement required. The results indicated that, with parental support, all items were generally understandable. The scale demonstrated excellent reliability in our sample (Cronbach’s *α* = 0.83–0.91), supporting its application with this population. While pediatric‐specific measures such as the Problem Areas in Diabetes‐Teen (PAID‐T) and PAID‐Child (PAID‐C) can screen for overall distress, the DDS was chosen because it assesses distress across multiple specific domains. This facilitates identifying which aspects of diabetes distress are most problematic, helping with targeted interventions.

Diabetes self‐management was evaluated using two key subscales from the Chinese adaptation of the Self‐Management of Type 1 Diabetes in Adolescents (SMOD‐A) scale [25], originally developed in English by Schilling et al. [26]. The Care Activities subscale (15 items) measured the frequency of daily diabetes management behaviors, while the Problem Solving subscale (seven items) assessed adaptive decision‐making capabilities. Both subscales used a four‐point Likert scale (0 = never, 1 = sometimes, 2 = most of the time, 3 = always), with higher composite scores indicating better self‐management skills. The Cronbach’s alpha values were 0.78 and 0.83, respectively.

Quality of life was measured using the Chinese version of the Pediatric Quality of Life Inventory (PedsQL) 3.2 Diabetes Module [27], originally developed by Varni et al. [28]. This 33‐item instrument provides a comprehensive assessment of diabetes‐specific quality of life across multiple domains. Responses were transformed to a 0–100 scale, with higher scores reflecting better quality of life. In this study, the Cronbach’s alpha values of the five subscales ranged from 0.85 to 0.92.

### 2.6. Data Collection

This study examined how diabetes distress, quality of life, diabetes care activities, and problem‐solving relate to self‐management in Chinese school‐aged children with T1DM during the pandemic. The study was carried out between June and December 2022 using the Survey Star digital platform (Changsha Ran Xing Science and Technology, Changsha, China). Participants accessed the questionnaires via unique QR codes, and survey links were also distributed through the official WeChat accounts of the participating institutions, which collectively reached an estimated audience of 50,000 individuals. All three institutions involved are members of the China Alliance for type 1 Diabetes and adhere to its standardized patient service protocols.

In China, WeChat is a widely utilized platform among the general population. At these centers, diabetes education nurses regularly encourage patients and their families to join the respective center’s WeChat group during outpatient visits. This practice serves as a primary channel for disseminating information about national diabetes‐related public activities and resources, effectively reaching most families seeking care at these clinics. While recruitment through WeChat may introduce the potential for self‐selection bias, this standardized approach enhances the likelihood of broad representation within the patient population.

To ensure data completeness and quality, all questionnaires were designed to require mandatory responses for each item prior to submission. Parents or guardians supervised the response process to facilitate understanding and accuracy, although this could introduce some level of social desirability bias.

### 2.7. Statistical Analyses

The data were double‐entered and analyzed using the IBM SPSS Statistics for Windows 22 (IBM Corp., Armonk, NY, USA). Descriptive statistics were used for categorical variables, such as sex, maternal education level, paternal education level, annual family income, and insulin treatment regimen, and for continuous variables, such as age, diabetes duration, and HbA1c level. Pearson correlation coefficients were calculated to evaluate the associations between the four domains of diabetes distress and quality of life.

Structural equation modeling was conducted using the Mplus program, version 7.4 (Muthén and Muthén, Los Angeles, CA, USA). Coefficients and standard errors were calculated, adjusting for age, sex, diabetes duration, HbA1c level, and insulin treatment regimen (categorized as <4 daily injections versus ≥4 daily injections or insulin pump use), for the direct and indirect (mediation) effects of the independent variables on the dependent variable. Model fit was assessed using the comparative fit index (CFI), the Tucker–Lewis index (TLI), and root mean square errors of approximation (RMSEA). A CFI and TLI greater than 0.9 and an RMSEA less than 0.05 were considered to indicate an acceptable model fit [29].

## 3. Results

### 3.1. Sociodemographic and Clinical Characteristics of the Participants

The mean age of the participants was 9.6 (standard deviation [SD] 1.6) years, and 68.3% of the participants were male. In terms of parental education, 83.3% of the mothers and 77.1% of the fathers had more than 9 years of education. One‐tenth (10.0%) of the children were treated with insulin pumps. The mean diabetes duration was 3.3 (SD 2.6) years, and the mean HbA1c level was 8.5% (SD 3.0%). The participants’ characteristics are shown in Table 1.

**Table 1:** Sociodemographic and clinical characteristics of the participants (*n* = 341).

Characteristics	*n* (%)	Mean (SD)
Sociodemographic variables		
Age (years)	—	9.6 (1.6)
Gender		
Male	233 (68.3%)	—
Female	108 (31.7%)	—
Mother’s education level		
<9 years	57 (16.7%)	—
≥9 years	284 (83.3%)	—
Father’s education level		
<9 years	78 (22.9%)	—
≥9 years	263 (77.1%)	—
Family annual income		
Less than US$3038 (low‐income) group	24 (7.0%)	—
US $3038–$6076 (middle‐income) group	51 (15.0%)	—
US $6076+ (high‐income)	266 (78.%)	—
Clinical characteristics		
Insulin treatment regimen		
<4x per day	87 (25.5%)	—
≥4x per day	220 (64.5%)	—
Insulin pump	34 (10.0%)	—
Diabetes duration (years)	—	3.3 (2.6)
HbA1c (%)	—	8.5 (3.0)

*Note:* China’s National Bureau of Statistics points out that the low‐income group refers to the group whose family annual income is less than 20,000 RMB (US$3038); the middle‐income group refers to the group whose family annual income is 20,000–40,000 RMB (US$3038–US$6076); the higher or high‐income group refers to the group whose family annual income is more than 40,000 RMB (US$6076+, China’s National Bureau of Statistics, 2017).

### 3.2. Descriptive Statistics of Diabetes Distress Levels, Care Activities and Problem‐Solving Related to Diabetes Self‐Management, and Quality of Life

The total DDS scores ranged from 1 to 6, and the mean total score was 3.54 (SD 1.32). The mean DDS subscale scores were as follows: Emotional Burden, 3.56 (SD 1.32); Physician‐Related Distress, 3.57 (SD 1.42); Regimen‐Related Distress, 3.53 (SD 1.35); and Diabetes‐Related Interpersonal Distress, 3.51 (SD 1.47). The scores of all subscales ranged from 1 to 6. The mean Diabetes Care Activities score was 24.77 (SD 4.92), ranging from 11 to 43, and the mean Diabetes Problem Solving score was 11.06 (SD 5.23), ranging from 0 to 21. The mean PedsQL 3.2 Diabetes Module (quality of life) score was 51.63 (SD 24.31), ranging from 5.17 to 97.41.

### 3.3. Correlations Between the Four Domains of Diabetes Distress and Quality of Life

The four domains of diabetes distress (emotional burden, physician‐related distress, regimen‐related distress, and diabetes‐related interpersonal distress) exhibited significant negative correlations with quality of life, ranging from −0.44 to −0.77 (*p* < 0.01). The Durbin–Watson coefficient ranged from 1.12 to 2.03, and the VIF varied from 5.53 to 6.67, indicating no significant multicollinearity among these variables. The correlations between the four domains of diabetes distress and quality of life are displayed in Table 2.

**Table 2:** Correlations and descriptive statistics for quality of life by four domains of diabetes distress.

	Mean ± SD	Quality of life
Emotional burden	3.56 ± 1.32	−0.740 ^∗∗^
Physician‐related distress	3.57 ± 1.42	−0.741 ^∗∗^
Regimen‐related distress	3.53 ± 1.35	−0.767 ^∗∗^
Diabetes‐related interpersonal distress	3.51 ± 1.47	−0.744 ^∗∗^

^∗∗^Indicates significant (*p* < 0.01).

### 3.4. Mediation Analysis of the Effects of Diabetes Care Activities and Problem‐Solving on the Relationship Between Diabetes Distress and Quality of Life

**Figure 1:**
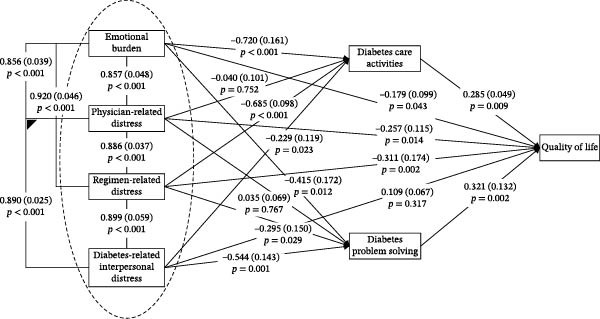
Mediation model for the effect of diabetes care activities and diabetes problem solving on the relationships between diabetes distress and quality of life. Standardized coefficients and errors terms for each path are provided. Significant associations are in bold typeface for emphasis.

The mediation model, which accounted for age, sex, diabetes duration, HbA1c level, and insulin treatment regimen, demonstrated an excellent fit to the data (CFI = 1.00, TLI = 1.00, RMSEA = 0.00). Fit indices showed that diabetes care activities and diabetes problem‐solving mediated the relationship between diabetes distress and quality of life. As shown by the pathways in Figure 1, diabetes care activities significantly related to most diabetes distress domains (emotional burden: *p* < 0.001; regimen‐related distress: *p* < 0.001; diabetes‐related interpersonal distress: *p* = 0.023). Similar significant links were observed between diabetes problem‐solving and most diabetes distress domains (emotional burden: *p* = 0.012; regimen‐related distress: *p* = 0.029; diabetes‐related interpersonal distress: *p* = 0.001). Conversely, neither diabetes care activities nor diabetes problem‐solving were significantly associated with physician‐related distress (both *p* > 0.05). Both diabetes care activities and diabetes problem‐solving were significantly associated with quality of life (*p* = 0.009 and 0.002, respectively). Additionally, all diabetes distress domains except diabetes‐related interpersonal distress (*p* = 0.317) had significant direct effects on quality of life (*p* = 0.002 to 0.043). Moreover, as shown in Table 3, the total effects of all four diabetes distress domains on quality of life were statistically significant (emotional burden: *β* = −0.161 [SD 0.085], *p* = 0.039; physician‐related distress: *β* = −0.181 [SD 0.085], *p* = 0.021; regimen‐related distress: *β* = −0.355 [SD 0.102], *p* = 0.001; diabetes‐related interpersonal distress: *β* = −0.091 [SD 0.065], *p* = 0.048).

**Table 3:** Direct and indirect (mediation) effects of diabetes distress on quality of life.

Variable	Effects	Diabetes distress			
		Emotional burden *β* (95% CI)	Physician‐related distress *β* (95% CI)	Regimen‐related distress *β* (95% CI)	Diabetes‐related interpersonal distress *β* (95% CI)
Quality of life	Total effect	**−0.161 (−0.248, −0.039)**	**−0.181 (−0.352, −0.136)**	**−0.355 (−0.534, −0.218)**	**−0.091 (−0.156** **, −0.023)**
	Indirect effect through diabetes care activities	**−0.108 (−0.190, −0.030)**	−0.002 (−0.081, 0.106)	**−0.272 (−0.520, −0.115)**	−0.043 (−0.087, 0.002)
	Indirect effect through diabetes problem solving	**−0.136 (−0.215, −0.040)**	−0.040 (−0.092, 0.017)	**−0.175 (−0.430, −0.039)**	**−0.166 (−0.295, −0** **.026)**
	Direct effect	**−0.193 (−0.317, −0.061)**	**−0.219 (−0.408, −0.091)**	**−0.311 (−0.503, −0.148)**	0.057 (−0.177, 0.271)

*Note:* Significant associations are in bold typeface for emphasis and were determined by a 95% bias‐corrected unstandardized bootstrapped confidence interval (based on 10,000 bootstrapped samples) that does not contain zero.

Significant indirect effects were observed, suggesting that the associations of both emotional burden and regimen‐related distress with poorer quality of life may operate through reduced engagement in diabetes care activities (Emotional Burden: 95% CI: −0.190, −0.030; Regimen‐Related Distress: 95% CI: −0.520, −0.115). The indirect effects of physician‐related distress (95% CI: −0.081, 0.106) and diabetes‐related interpersonal distress (95% CI: −0.087, 0.002) on quality of life via diabetes care activities were not statistically significant. The confidence intervals for both estimates included zero, indicating that the data do not provide sufficient evidence to support a mediating role of diabetes care activities in the relationships between these specific distress domains and quality of life.

Three domains of diabetes distress had significant indirect effects on quality of life through diabetes problem‐solving (emotional burden: 95% CI: −0.215, −0.040; regimen‐related distress: 95% CI: −0.430, −0.039; diabetes‐related interpersonal distress: 95% CI: −0.295, −0.026). Conversely, the indirect effect of physician‐related distress on quality of life via diabetes care activities was not statistically significant (95% CI: −0.092, 0.017). Since the confidence interval includes zero, the data do not establish this mediational pathway.

## 4. Discussion

This study found that diabetes problem‐solving slightly but significantly lessens the negative impact of diabetes distress on quality of life, more effectively than care activities. These results are not congruent with previous findings suggesting that diabetes care activities are at the core of self‐management behavior for individuals with T1DM [19]. Our findings also indicate that neither diabetes care activities nor diabetes problem‐solving mediated the negative relationship between physician‐related distress and quality of life. These findings suggest that interventions should focus on improving diabetes‐related care activities and problem‐solving skills to reduce the negative effects of emotional burdens and regimen‐related distress on quality of life.

Unlike a survey of a similar population conducted before the COVID‐19 lockdowns [30], this study indicates elevated diabetes distress levels in all four domains. This is in line with a Spanish study that reported heightened psychological distress among children with autism spectrum disorder during a COVID‐19 lockdown [31]. This surge in distress can be attributed to increased uncertainty and fear caused by the pandemic. Factors such as school closures, quarantines, social isolation, heightened family stress levels, limited peer interactions, and difficulties in accessing healthcare may contribute to increased diabetes distress among school‐age children with T1DM [32].

Notably, the mediating effects of diabetes care activities and problem‐solving on the relationship between diabetes distress and quality of life remained significant, even though the sample reported clinically elevated levels of diabetes distress. This finding indicates that even in high‐distress situations—such as during the COVID‐19 pandemic—individual differences in self‐management behaviors play a crucial role in determining quality of life. Specifically, those who maintained higher levels of self‐management, even while experiencing significant distress, reported better quality‐of‐life outcomes. Several factors may contribute to this, including personal resilience, strong social support networks, and the quick adoption of telehealth services, all of which may have lessened the usual negative impact of distress on self‐management behaviors [33].

Our findings also suggest that enhanced diabetes care activities mitigated the adverse impacts of emotional burdens and regimen‐related distress on quality of life. In the context of T1DM, emotional burdens encompass nonpsychiatric emotional reactions to the onset and course of T1DM, including perceived self‐management demands and the fear of diabetes‐related complications [34]. Similarly, regimen‐related distress arises from the continuous T1DM self‐management demands [14]. Although its association is weak, adequate performance of diabetes care activities may partially assist school‐age children in managing emotional burdens and regimen‐related distress, potentially leading to slight improvements in quality of life.

This study found that diabetes problem‐solving slightly mediated the negative impact of emotional and regimen‐related distress on quality of life. Diabetes problem‐solving entails decision‐making processes related to diabetes care, particularly in terms of adjusting treatment regimens [17]. Resolving diabetes‐related problems enables school‐age children to actively engage in various activities, including social and schooling activities, alleviate their worries about their health, and ultimately improve their quality of life [35]. Moreover, advanced problem‐solving skills, such as adjusting insulin doses based on carbohydrates, exercise, and glucose monitoring, enable school‐age children to meet the constant T1DM self‐management demands [26]. Thus, such skills play a pivotal role in alleviating regimen‐related distress, contributing to a better quality of life.

Interestingly, unlike diabetes care activities, diabetes problem‐solving played a mediating role in mitigating the negative association between increased diabetes‐related interpersonal distress and poor quality of life. Diabetes‐related interpersonal distress may stem from unsupportive relationships or may be triggered by the impact of T1DM on interpersonal goals [36]. For instance, when confronted with conflicting schedules or encouraged by peers to eat inappropriate foods, school‐age children with T1DM may experience diabetes‐related interpersonal distress [14]. School‐age children with good diabetes problem‐solving skills can adjust their insulin dosages based on their diets and exercise, thereby attaining more stable blood sugar levels and a good quality of life. Therefore, such skills can minimize the negative effects of interpersonal‐related distress on quality of life.

In this study, we observed no mediating effects of diabetes care activities or diabetes problem‐solving on the relationship between physician‐related distress and quality of life. Physician‐related distress may result from unresponsive healthcare providers or from difficulties in accessing healthcare services [14]. During the pandemic, school‐age children with T1DM may have visited hospitals less frequently, leading to fewer opportunities for communication with physicians [37]. Consequently, the observed nonsignificant mediating roles of diabetes care activities and diabetes problem‐solving in the relationship between physician‐related distress and quality of life.

### 4.1. Limitations

One of the key strengths of our study was the inclusion of participants from 16 different provinces across China, which provided a diverse and representative sample. However, it is also important to acknowledge several limitations of this study. First, the cross‐sectional design limits our ability to draw causal conclusions from the observed relationships, meaning we cannot definitively establish whether one variable influences another. Second, data collection during the COVID‐19 pandemic may limit its generalisability over time. Third, nonstratified sampling based on geographic, socioeconomic, or demographic factors can introduce potential selection biases. Fourth, although pediatric‐specific measures (e.g., PAID scales) are well‐validated for screening overall distress, the DDS was selected for its ability to identify key dimensions to inform precise interventions. Additionally, WeChat‐based recruitment may overrepresent digitally engaged, health‐conscious families, while parental supervision during questionnaire completion could introduce social desirability bias. Furthermore, the online methodology may exclude populations with limited internet access or lower digital literacy. Finally, although statistically significant, the small effect sizes (e.g., *β* < |0.3|) in mediation analyses suggest that these associations may have limited clinical significance without additional interventions targeting other psychosocial or biological factors.

### 4.2. Implications

Our study has several key implications for researchers and nurses working with school‐age children with T1DM. First, we advise that nurses routinely monitor diabetes distress levels in clinical practice. Second, interventions aimed at improving self‐management skills and diabetes‐related problem‐solving are recommended for this population at each clinic visit. Third, to reduce the adverse effects of physician‐related distress on quality of life, it is essential for nurses to actively communicate with children with T1DM and emphasize the importance of psychological well‐being during routine follow‐ups. Fourth, future research could explore other potential mediators, such as symptoms of depression and peer relationships, in the link between physician‐related distress and quality of life. Finally, the core self‐management strategies identified are likely to benefit this population not only in routine care but also during future large‐scale public health emergencies.

## 5. Conclusions

Our cross‐sectional results suggest that, compared to diabetes care activities, problem‐solving related to diabetes self‐management may be linked to reduced diabetes distress across multiple areas (emotional burden, regimen‐related distress, and interpersonal distress) and their potential impacts on quality of life. While these observed associations cannot establish causal relationships due to the study design, they highlight the potential value of interventions targeting both diabetes care activities and problem‐solving skills for school‐age children experiencing distress. Future longitudinal studies are needed to examine the causal pathways between these factors and to investigate why neither approach appeared to mitigate physician‐related distress in our sample.

## Author Contributions

Jiaxin Luo and Qingting Li analyzed the data and wrote the manuscript. Jia Guo conceptualized this study, is the guarantor of this work, and had full access to all data, taking responsibility for data integrity and analysis accuracy. Jia Guo and Robin Whittemore contributed to the discussion and provided statistical analysis. Yuwen Gao, Fang Liu, Jie Zhong, and Ka Yan Ho reviewed the data and provided critical input during discussions, multiple revisions, and the final manuscript.

## Funding

This study was supported by the Noncommunicable Chronic Diseases‐National Science and Technology Major Project (Grant 2023ZD0508200, 2023ZD0508204), the National Natural Science Foundation of China (Grant 72264037), and the Sinocare Diabetes Foundation (Grant 2024SD05).

## Disclosure

The sponsors were not involved in the study design, data collection, analysis, or interpretation. They received the draft manuscript for informational purposes, but neither wrote it nor decided to submit it. All authors approve the final version as submitted. After using Grammarly tool, the authors reviewed and edited the content as needed and take full responsibility for the content of the published manuscript.

## Conflicts of Interest

The authors declare no conflicts of interest.

## Supporting Information

Additional supporting information can be found online in the Supporting Information section.

## Supporting information


**Supporting Information** Supporting File 1 includes the reporting checklist for the cross‐sectional study.

## Data Availability

The data that support the findings of this study are available from the corresponding author upon reasonable request.
